# Supplementary material for: The Protective Effects of Mcl-1 on Mitochondrial Damage and Oxidative Stress in Imiquimod-Induced Cancer Cell Death
Source: Cancers (Basel). 2024 Sep 2;16(17):3060. doi: 10.3390/cancers16173060 (PMC11394135; doi:10.3390/cancers16173060)
Supplement: Supplementary file 1 [file cancers-16-03060-s001.zip › cancers-3171665-supplementary.pdf]

A

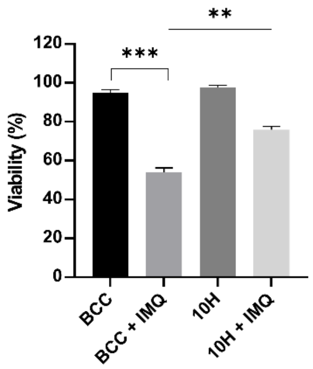

B

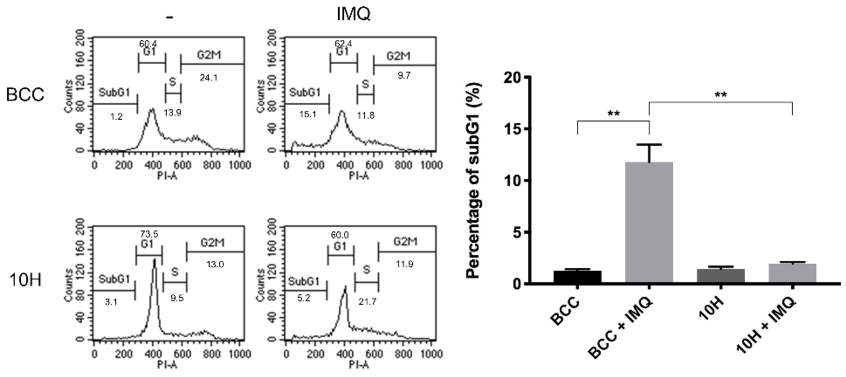

Supplementary Figure S1, (A) Mcl-1 overexpression attenuated IMQ-induced cell death in 10H cells. (B) Mcl-1 overexpression in 10H cells reduced the IMQ-induced apoptosis rate. Cells were treated with 50 µg/ml IMQ for 24 hours. After IMQ treatment, Cell viability was analyzed with trypan blue staining assay (A). Cell cycle analysis was performed by PI staining and flow cytometry (B). The results were statistically analyzed by two-way ANOVA. *P***<0.01 and *P****<0.01.

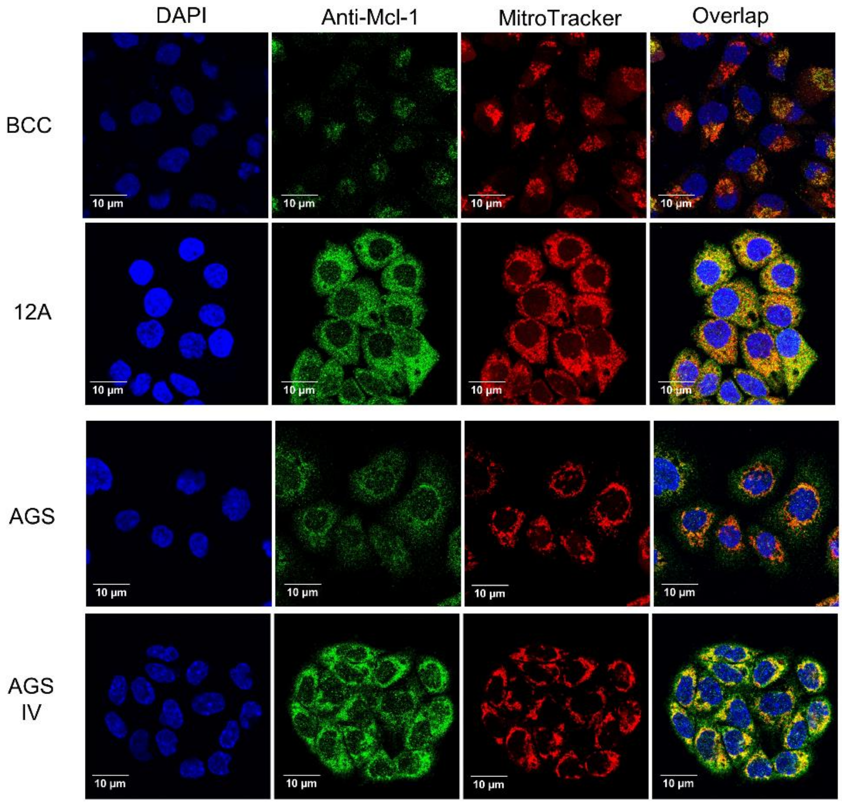

Supplementary Figure S2, The rate of colocalization of Mcl-1 and mitochondria was greater in Mcl-1-overexpressing cells. Mcl-1 overexpression increased the Mcl-1 localization rate to mitochondria in cancer cells. BCC control, BCC 12A, AGS control and AGS IV cells were treated with 50 µg/ml IMQ for 24 hours. After IMQ treatment, the cells were stained with 100 nM MitoTracker Red CMXRos for 30 min and then fixed with 1% paraformaldehyde overnight. After fixation, the cells were stained with a rabbit anti-Mcl-1 antibody overnight and then stained with a goat anti-rabbit antibody. Then, images were taken with a confocal microscope. Scale bars, 10 µm.

A

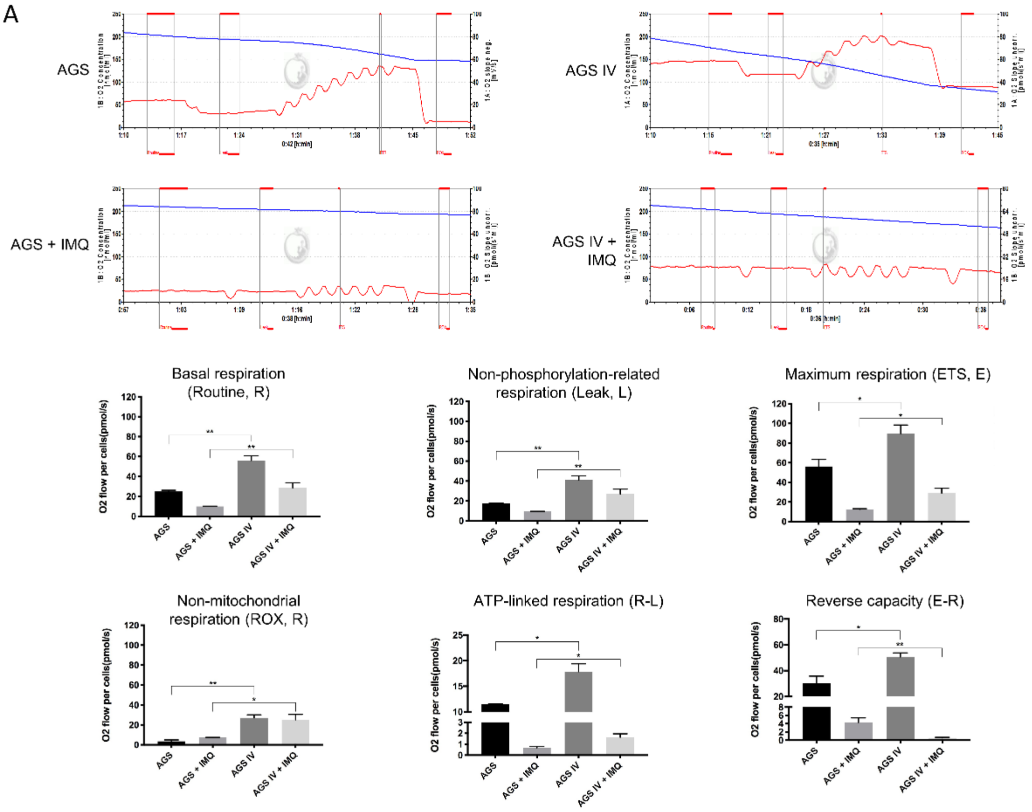

B

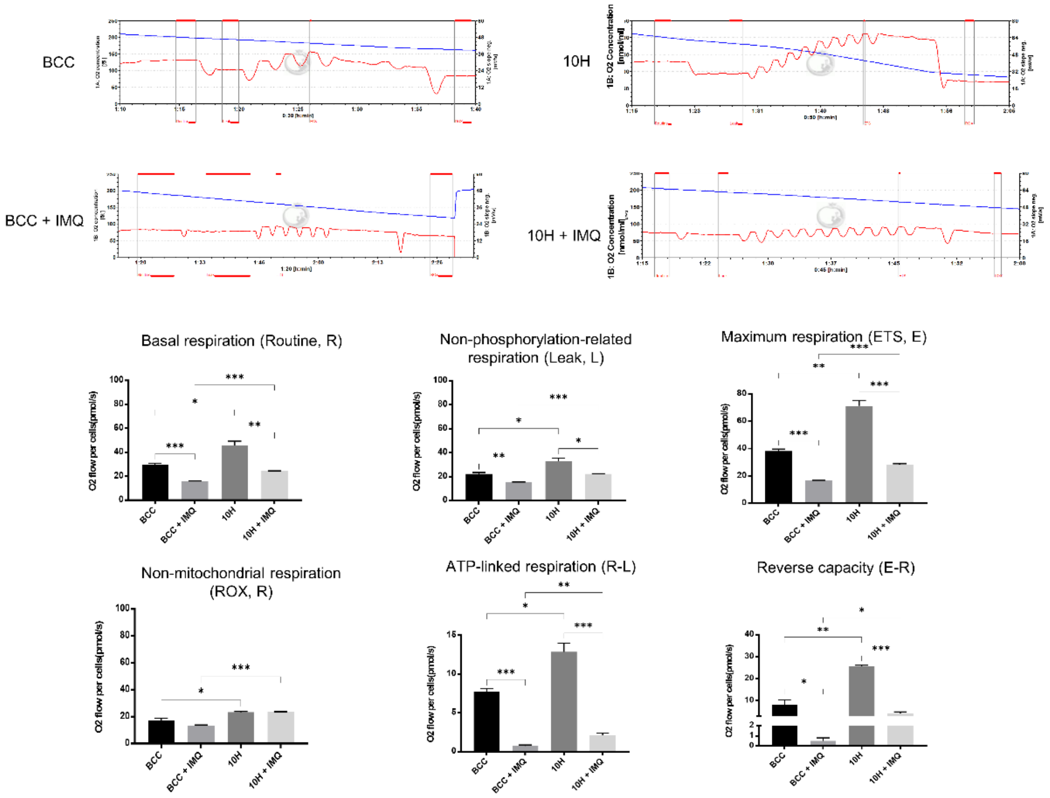

Supplementary Figure S3. Mcl-1 overexpression increased the mitochondrial oxygen consumption rate but partially counteracted the IMQ-mediated inhibition of the

mitochondrial oxygen consumption rate in AGS (A) and BCC (B) cells. AGS control, AGS IV cells, BCC control and 10H cells were treated with 50 μg/ml IMQ and sequentially treated with 2 μM oligomycin, 0.5 μM FCCP (titration concentration), 2 μM antimycin A1 and rotenone; the differences in mitochondrial OCRs were subsequently evaluated with an O2k respirometer. The data are expressed as the mean ± S.E.M. of three independent experiments. The statistical results were analyzed by two-way ANOVA. *P**<0.05 and *P***<0.01.

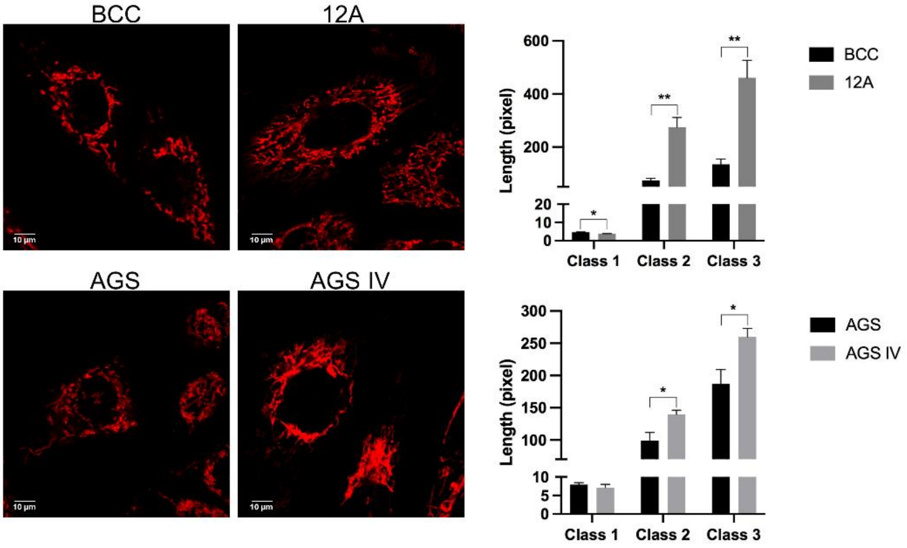

Supplementary Figure S4. Mcl-1-overexpressing cells contained more long and tubular mitochondria than their control counterparts. BCC control, BCC 12A, AGS control and AGS IV were stained with 100 nM MitoTracker Red CMXRos for 30 min before confocal imaging. Mcl-1 overexpression decreased mitochondrial fission but increased mitochondrial fusion in cancer cells. The mitochondrial morphologies were observed by confocal microscopy and analyzed by CellSens Software (Olympus). Scale bars, 10 μm. Class 1 is fragmented mitochondria. Class 2 and Class 3 are intermediate and tubular mitochondria, respectively. The results were statistically analyzed by one-way ANOVA. *P**<0.05 and *P***<0.01.

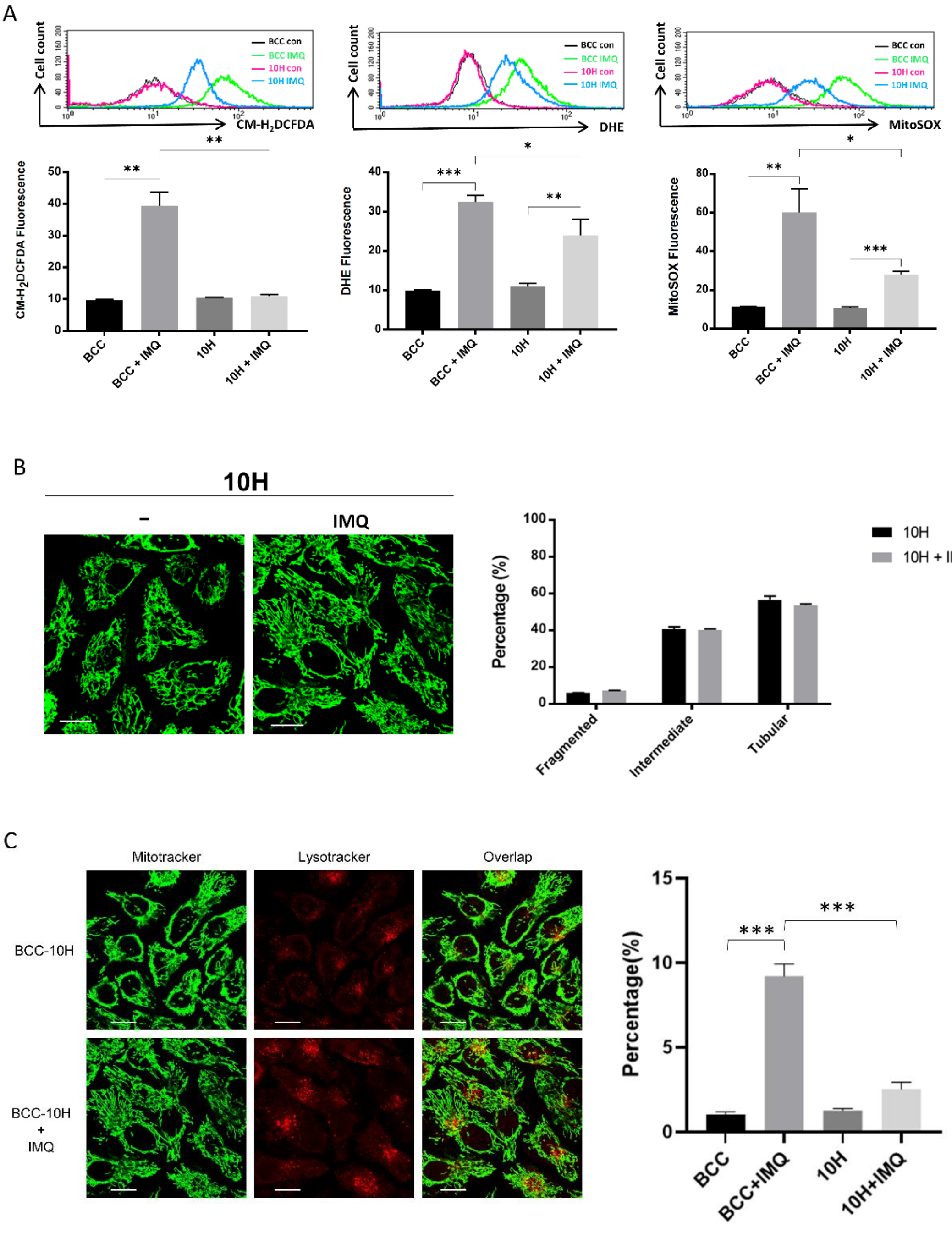

Supplementary Figure S5. (A) Mcl-1 overexpression attenuated IMQ-induced oxidative stress in 10H cells. (B) Mcl-1-overexpression abrogated IMQ-induced mitochondria fragmentation in 10H cells. (C) Mcl-1 overexpression modulated IMQ-induced mitophagy in 10H cells. 10H cells were treated with 50 μg/ml IMQ for 4 hours (A) or 24 hours (B and C). (A) The total ROS level, cellular superoxide and mitochondrial superoxide were measured by CM-H2DCFDA, DHE and MitoSOX staining via flow cytometry. (B) For mitochondrial morphology, the 10H cells were stained with 100 nM MitoTracker Green FM for 30 min, observed by confocal microscopy, and the number of mitochondria in each morphological class was

determined by CellSens Software (Olympus). Scale bars, 10 μm. (C) Cells were preincubated with 100 nM MitoTracker Green FM for 30 min followed by 50 μg/ml IMQ treatment for 24 hours and then stained with 1 μM LysoTracker Red DND-99 for 30 min. The colocalization rates of mitochondria with lysosomes was monitored by confocal microscopy and analyzed with Olympus FV10-ASW V4.2 software (Olympus). Scale bars, 10 μm. The data are expressed as the mean ± S.E.M. of three independent experiments. The statistical results were analyzed by two-way ANOVA. *P**<0.05, *P***<0.01 and *P***<0.001.
